# Data Analytics for Predicting Situational Developments in Smart Cities: Assessing User Perceptions

**DOI:** 10.3390/s24154810

**Published:** 2024-07-24

**Authors:** Alexander A. Kharlamov, Maria Pilgun

**Affiliations:** 1 Institute of Higher Nervous Activity and Neurophysiology, Russian Academy of Sciences, 117486 Moscow, Russia; kharlamov@analyst.ru; 2 Department of Intelligent and Information Systems and Technologies, Moscow Institute of Physics and Technology, 141701 Moscow, Russia; 3 Department of Software Engineering, Higher School of Economic, 109028 Moscow, Russia; 4 Department of Applied and Experimental Linguistics, Moscow State Linguistic University, 119034 Moscow, Russia; 5 Research Institute of Prospective Directions and Technologies, Russian State Social University, 129226 Moscow, Russia; 6 Department of General and Comparative-Historical Linguistics, Lomonosov Moscow State University, 119991 Moscow, Russia

**Keywords:** data analytics, predictions, social media, big data, smart city, urban project, perception

## Abstract

The analysis of large volumes of data collected from heterogeneous sources is increasingly important for the development of megacities, the advancement of smart city technologies, and ensuring a high quality of life for citizens. This study aimed to develop algorithms for analyzing and interpreting social media data to assess citizens’ opinions in real time and for verifying and examining data to analyze social tension and predict the development of situations during the implementation of urban projects. The developed algorithms were tested using an urban project in the field of transportation system development. The study’s material included data from social networks, messenger channels and chats, video hosting platforms, blogs, microblogs, forums, and review sites. An interdisciplinary approach was utilized to analyze the data, employing tools such as Brand Analytics, TextAnalyst 2.32, GPT-3.5, GPT-4, GPT-4o, and Tableau. The results of the data analysis showed identical outcomes, indicating a neutral perception among users and the absence of social tension surrounding the project’s implementation, allowing for the prediction of a calm development of the situation. Additionally, recommendations were developed to avert potential conflicts and eliminate sources of social tension for decision-making purposes.

## 1. Introduction

The development of smart cities is a significant issue that is receiving extensive coverage and active advancement in both scientific research and practical projects. Researchers discuss the socioeconomic, environmental, and policy implications of smart cities [1]; urban informatics for smart cities; optimization, smart industry, and smart public services; the Internet of Things; computational intelligence and urban informatics for smart cities; and innovative informatic approaches for smart cities [2]. They explore the architecture, design, and implementation of critical components of Sustainable Smart Cities to support governance, transportation, energy, healthcare, factories, technologies, security, agriculture, and education [3]. The authors believe that various technologies in a smart city will mitigate challenges and promote sustainable growth and future prospects for urban environments [4]. In addition, developing a smart city requires the creation of a model for scenarios and a corresponding web-based open-source tool for scenario editing. This tool provides a flexible solution capable of addressing various domains [5].

Recent scientific reflections and operational issues on the topic of “Smart City” have seen significant development. A multidisciplinary collective of researchers has summarized current challenges in designing the city of the future, highlighting new research directions in home infrastructure, small smart cities, energy transitions, connectivity, digitalization, and autonomous and connected mobility. They developed a model of urban development that integrates technical and humanistic cultures, fostering an open dialogue between various stakeholders [6].

A growing urban population leads to new challenges in metropolitan areas. Smart City technologies enable city authorities to develop effective solutions to ensure a high quality of life for citizens. Social media can be regarded as a real-time social sensor capable of sensing and collecting diverse information. This information, when combined with sentiment analysis, can enhance IoT sensors by providing user-demanded favorable data within smart systems [7]. In the context of smart cities, real-time social media data can be leveraged to identify and analyze citizen opinions. This data-driven approach allows for informed decision-making, predictions, and evaluations of various scenarios related to urban planning projects.

In particular, the development of the city’s transport system often encounters issues related to encroaching on the historically established environment and the habitual living conditions of its residents. These changes, even if vital for the city’s growth, can be perceived negatively by the populace. Proper and timely analysis of citizens’ opinions enables the formulation of compromise solutions and the development of recommendations to mitigate potential conflicts and ensures sustainable and smart urban development.

Research already exists, which analyzes various aspects of the development of transport systems in smart cities. A comprehensive study at the intersection of urban planning, transportation, technology, and smart city development is presented in [8]. In a survey of AI, Torre-Bastida analyzed the latest research efforts revolving around big data for the transportation and mobility industry [9], an overview of data models, standards, and their relationships presented in [10].

However, methods for determining citizens’ opinions in real time regarding the implementation of urban development projects have yet to be fully explored in the scientific literature. It is evident that solutions aimed at urban development in the context of “smart” cities should have the support of citizens and align with their vision of a comfortable urban environment.

The aims of this study are as follows:

1. Development of algorithms for analyzing and interpreting social media data to assess citizens’ opinions in real time.
2. Development of algorithms for verifying and examining social media data for analyzing social tension and predicting the development of situations during the implementation of urban projects.
3. Testing the developed algorithms using the example of implementing an urban project in the field of transportation system development.

### 1.1. Related Work

#### 1.1.1. Data Analytics

Data analytics is one of the most in-demand fields of scientific research. Researchers studied the background to and the concepts of big data, big data analytics, and cloud computing; the process of setting up, configuring, and getting familiar with the big data analytics working environments; big data processing systems—from installing these systems to implementing real-world data applications, along with the necessary codes; and the details of big data storage technologies, including their types, essentiality, durability, and availability, which reveal their differences in their properties [11]. Various aspects of data analytics are presented in numerous studies: a theoretical and practical view on data analytics from the EDISON project (Education for Data Intensive Science to Open New science frontiers) [12]; forecasting, data analytics, mathematics for data science, graph theory and application in data science, data visualization, computer vision, and analytics for social networks [13]; innovations in data analytics [14]; an in-depth analysis of successful data-driven initiatives, highlighting how organizations have leveraged data to drive decision-making processes, optimize operations, and achieve remarkable outcomes [15]; data management, analytics and innovation [16]; data analytics, data management, big data, computational intelligence, and communication networks [17]; integrating data analytics in system engineering [18]; security, privacy and data analytics [19].

However, it should be noted that data analytics for predicting the development of situations in smart cities still requires further development.

#### 1.1.2. Predictive Analytics

The development of electronic communications and social networks has created big data of unique origin, which allows for solving various problems in real time and for predictive analytics.

In recent studies, data-based prediction has been widely used, including data acquisition methods and sources, exploratory data analysis and sensitivity analysis, data-based modeling for prediction, data-based modeling for monitoring and control, and data-based optimization of processes [20].

Real-time data analysis is also in demand in a variety of areas: in the field of medical laboratory diagnostics [21]; for observations of coupled biological, chemical, and physical processes in the ocean from the macro to micro scale [22]; in real-time intelligent systems [23]; for high-speed 3d railroad tie deflection mapping in real time using an array of air-coupled non-contact transducers [24]. The integration of CPS with IoT and big data analytics, modeling solutions, distributed management, efficient energy management, cyber–physical systems research, and education with applications in industrial, agriculture, and medical domains were analyzed in detail [25]. An exemplary comparison of the pre-processing techniques, Convolutional Neural Network models, frameworks, and optimization techniques applied to prediction, which detect and classify plant diseases using leaf images as a dataset, is presented in [26]. According to researchers, in today’s economy, businesses simply cannot be competitive without engaging big data in one way or another, in support of operations, management, planning, or simply basic hiring decisions [27].

It is evident that predicting the development of situations in smart cities is crucial for promoting sustainable, inclusive, and smart urban development.

#### 1.1.3. Social Media Data Analysis

The expansion of virtual communication and the growth of social media has led to the emergence of a vast amount of research reflecting various aspects of online interaction. It should be noted that Reda Alhajj and Jon Rokne analyzed fundamental concepts and research directions in the areas of social networks and applications to data mining [28].

The specifics of information dissemination and perception in social networks have already received multidimensional attention in scientific research [29,30].

Researchers have analyzed various aspects of virtual communications: the language of social media, identity, and community on the Internet [31]; interpersonal and intergroup communication characteristics [32]; clustering [33]; barriers in online communication [34,35]; maximizing influence in social networks [36,37]; analytics in social media platforms and the web [38]; integral communication and digital identity [39], and others.

Perception issues also have a long-standing tradition of study across various disciplinary fields.

Csaba Benedek selected problems of machine perception, using various 2D and 3D imaging sensors, proposed several new original methods, and also provided a detailed state-of-the-art overview of existing techniques for automated, multi-level interpretation of the observed static or dynamic environment [40].

The researchers analyzed a number of new and traditional issues pertaining to the roles of representations in visual perception; the issue of whether the perceptual state, like its distal objects, is structured, for instance, by possessing a spatial character [41].

An important update on the current state of research in speech perception, including reviews of contemporary developments in the auditory cognitive neuroscience of speech perception—counting both behavioral and neural contributions—is shown in [42].

In the scientific tradition, numerous studies have examined various aspects of speech perception in its multi-level processing system. For example, subjective perception features were analyzed in [43], individual differences and the interaction of perception and speech production in [44], deception perception by bilinguals in [45], and perception and processing of speech by blind bilinguals and monolinguals in [46]. Recently, special attention has been paid to issues of perception transformation in the virtual environment [47].

The use of neural systems’ potential for speech perception and production analysis has also been present in scientific research for quite some time. For example, in Stefan Uhrig’s study, a process-oriented approach to speech quality assessment was used, applying subjective, behavioral, and neurophysiological levels of analysis, which revealed the impact of speech transmission quality on human information processing [48].

Meanwhile, psycholinguistic methodologies and neural network approaches are not yet widely applied enough to study the perception of social media content, particularly for analyzing how users perceive specific situations and identifying social tensions and well-being.

The algorithms for validating and studying social network data require further refinement and adaptation to specific practical projects. It is evident that the analysis and interpretation of large volumes of social network data require an interdisciplinary approach, integrating methods from various disciplines (computer science, mathematics, statistics, linguistics, psycholinguistics, sociology, psychology, etc.). The combination of scientific paradigms should likely be determined by the specific goals and tasks of a particular project.

## 2. Materials and Methods

### 2.1. Data

The research focused on analyzing the development of the situation related to the first phase of implementing a major urban development project in the transport sector of a metropolis. Moscow is the largest transport hub in Russia. Public transport plays an important role in the city’s transport system, with more than 16.5 million trips made per day (about 6 billion trips per year). The city’s development requires the transformation of the road transport system. The construction of the project analyzed in this study began in 2021 and should be completed by 2030. The project’s implementation is expected to improve the transport situation for more than 800 thousand residents of five city districts.

The material for the study included data from social networks, messenger channels and chats, video hosting platforms, blogs, microblogs, forums, and review sites. Table 1 presents the quantitative characteristics of the database. Users who received information about the project were involved in the discussion of the project implementation and their activity, the indicator of growth, and retention of attention around the project (Table 1; Table S1).

Particular attention was paid to data from the video hosting platform YouTube, the social network VKontakte, the messenger Telegram, and the service Dzen.

**Figure 1:**
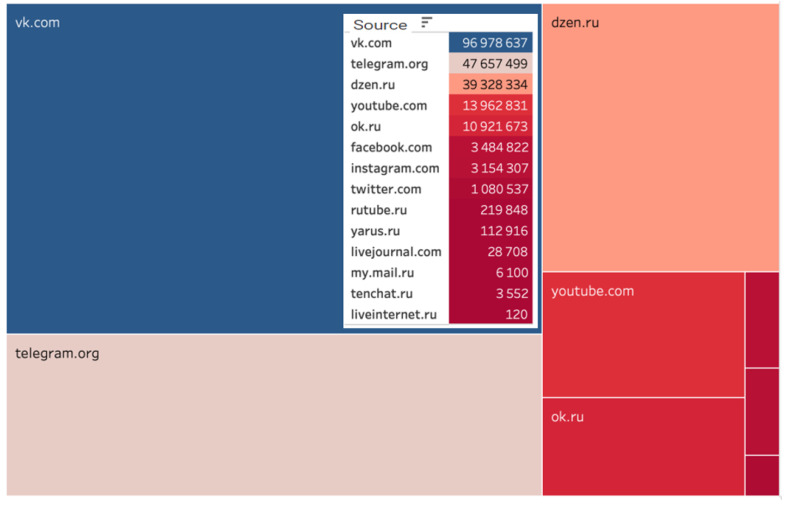
Digital platforms on which relevant content was generated.

The data analysis revealed that actors preferred the specified digital platforms when generating content related to the implementation of the analyzed project. The leader in quantitative metrics was the social network VKontakte. The subsequent rankings were Telegram, Dzen, and YouTube (Figure 1).

The data collection was carried out using the Brand Analytics “https://brandanalytics.ru (accessed on 31 July 2023)” monitoring and analysis system for social networks.

The data were gathered from 28 August 2022, 00:00, to 31 July 2023, 23:59.

Table 2 presents the quantitative characteristics of the leading digital platforms where users generate relevant content. VKontakte is the most popular social network in the Russian-speaking segment of the Internet. Telegram has become the second most popular messenger for discussing project implementation, with its popularity attributed to the user-friendly interface and security features. The third position in the ranking is held by Dzen, a Russian blogging platform for creating and viewing content, originally launched by Yandex in June 2015 and owned by VKontakte since September 2022. The fourth place is occupied by the video hosting platform YouTube (see Table 2).

**Figure 2:**
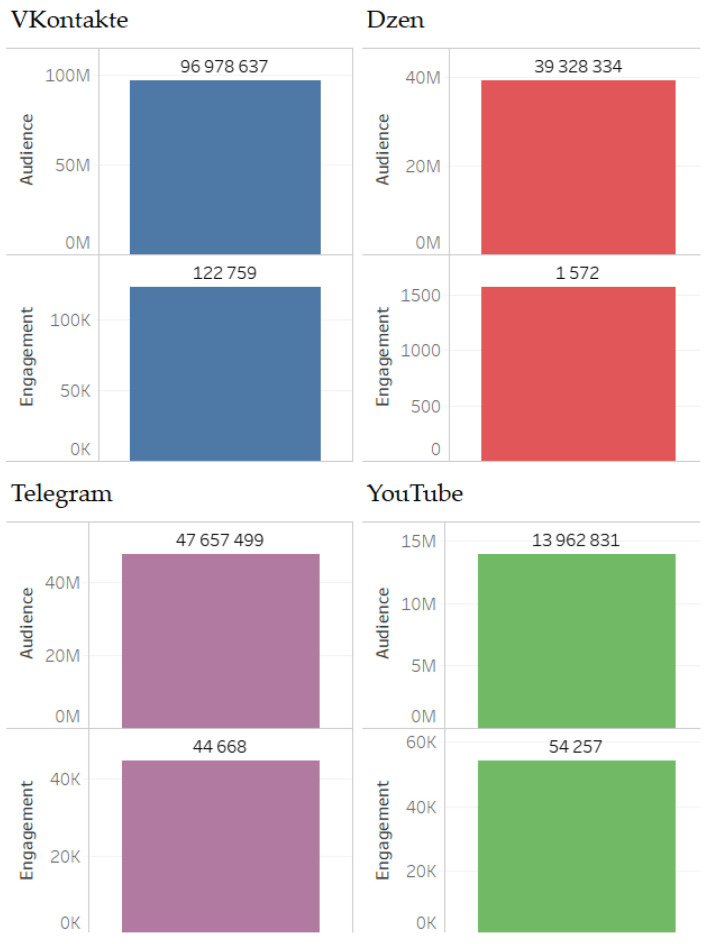
Main digital platforms on which relevant content was generated.

In accordance with this rating, indicators of the audience and engagement of the digital platforms on which the relevant content was generated are distributed (Figure 2).

### 2.2. Method

The study utilized an interdisciplinary approach to analyze large volumes of social media data [49].

To verify the research results and accomplish the study’s objectives, two algorithms were developed. Both algorithms include a preliminary stage involving data collection and cleaning, identification of various types of actors and digital platforms, as well as analysis of the audience and engagement.

In the first algorithm, the main part of the analysis is dedicated to sentiment analysis, followed by clustering of the database based on sentiment and a semantic analysis that involves forming a semantic network, identifying the semantic core, and interpreting the obtained data.

Sentiment analysis was conducted using the sentiment determination module of Eureka Engine, which employs a statistical algorithm for Conditional Random Fields (CRF) using sentiment dictionaries. Sequences of lexemes were used as input data, after which the algorithm calculates the probabilities of possible tag sequences and selects the most probable one.

The semantic network, formed as a set of interrelated concepts, was created using a model with neural-like elements featuring temporal summation of signals or corticomorphic associative memory. Extraction of the semantic core (nominations with link weights of 98–100) and neural network text analysis allowed for identifying themes that garnered the most attention from actors and semantic accents that were most important to residents when discussing the project’s implementation issues.

Concept extraction block. The block for extracting key concepts of the subject area (words and phrases) is created on the basis of a software model of hierarchical structures of neurons with time summation of signals [50] (ANN), and it implements algorithms for the automatic formation of a frequency dictionary of the text.

The number of INS levels in the hierarchical structure determines the a priori specified maximum permissible length of a concept of the subject area and is equal to twenty.

The first level of the hierarchical structure presents a dictionary of two-letter special words of the subject area—words passed through all filters of the linguistic processor and not classified as commonly used, as well as two-letter combinations of words from this dictionary. The same place stores two-letter words of common vocabulary included in stable phrases and their initial two-letter fragments. The second level of the hierarchical structure is represented by ANNs storing dictionaries of three-letter words and letter combinations from dictionaries of special and common words encountered in the text in the form of indexes of elements of the corresponding first-level dictionaries supplemented by one more letter. At subsequent levels, the information representation is completely uniform—the ANN stores indexes of storage elements of the lower ANN level supplemented by one letter.

In the process of forming the information representation in the hierarchical structure from the ANN, the frequency of occurrence of each letter combination in the corresponding ANN elements is calculated. The frequency of words (letter combinations that do not have a continuation at the next level) is used for subsequent analysis.

The thus formed representation of the text vocabulary is then subjected to a threshold transformation by frequency of occurrence. The threshold reflects the degree of detail of the text description. In the process of statistical analysis, stable terms and terminological phrases are identified in the hierarchical structure of the ANN, which then serve as elements for constructing a semantic network. In this case, commonly used words as well as phrases containing only commonly used words are omitted.

Semantic network formation block. The semantic network formation block is implemented as a database in which semantic connections of the concepts of the subject area identified in the previous step are presented. Since the types of semantic connections [50] are not determined in the system, such connections are simply associative connections.

The frequency of their joint occurrence in one sentence is used as a criterion for determining the presence of a semantic connection between a pair of concepts. Exceeding a certain threshold by the frequency allows us to speak of the presence of an associative (semantic) connection between the concepts, and joint occurrences of concepts in sentences with a frequency less than the threshold are considered simply random.

**Figure 3:**
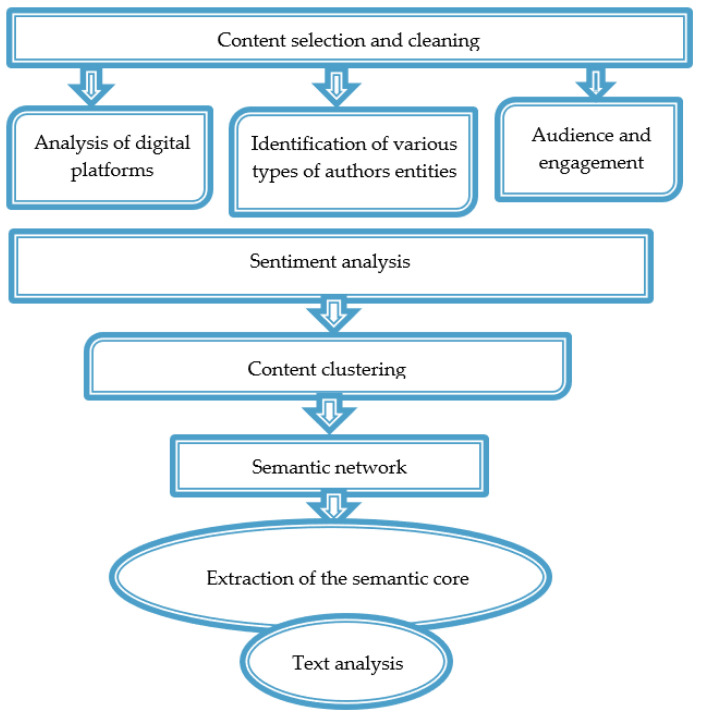
Algorithm 1.

Elements of the semantic (associative) network and their connections have numerical characteristics that reflect their relative weight in a given subject area—semantic weight. With a sufficiently representative set of texts describing the subject area, the frequency values of concept occurrences do indeed reflect the corresponding semantic (subjectively assessed) weights. However, for small training samples, particularly when analyzing a separate text, not all frequency characteristics correspond to the actual semantic weights—the importance of concepts in the text. For a more accurate assessment of the semantic weights of concepts, the weights of all concepts associated with them are used, i.e., the weights of the entire “semantic condensation”. As a result of such an analysis, the greatest weight is acquired by concepts that have powerful connections and are located, as it were, in the center of “semantic condensations” [50] (Figure 3).

The main part of the second algorithm was the analysis of aggression, the construction of an associative network, the analysis of lexical associations, and contexts with reactions.

The level of aggression is shown to be the most accurate indicator of the emotional perception of a situation. The dynamics of aggression levels, when present, are important for predictive analytics. Detecting aggression in user-generated content, especially its increase, serves as an indicator of a developing conflict situation and allows for forecasting the escalation of social tension. The calculation and dynamics of social stress and well-being indices serve as additional markers of the situation’s development. A decrease in the social stress index and an increase in the social well-being index clearly indicate a positive development of the situation.

The degree of aggression was identified based on the analysis of lexical tags of aggression determined by experts based on the analysis of the thematic structure and semantic and associative networks. The set $l_{i}$ of lexical tags, which form the lexical mask $L=\left\{l_{i}\right\},\;i=1\ldots I$, were automatically ranked within the analyzed text corpus by determining their semantic weight $r_{i}$ in this corpus. Summing the ranks of lexical tags weighted by their significance $w_{i}$ in this domain, as assigned by the expert, allows for the calculation of the integral degree of aggression present in the corpus texts: $W=\sum r_{i}w_{i}$ [50]. Moreover, the calculation of social stress and well-being indices and the analysis of their dynamics allow for monitoring the level of emotional tension and the perception of actors, as well as forecasting the development of the situation around the analyzed object. The author’s algorithm for determining aggression and calculating the social stress and well-being indices using large volumes of social media data are presented in [51].

The source of data for identifying and analyzing the indices of social stress and social well-being was a consolidated database, the quantitative characteristics of which are presented in Table 1.

When forming an associative network, the criterion for determining the presence of a connection between a pair of concepts is the frequency of their co-occurrence in a single sentence. If the frequency exceeds a given threshold, it indicates an associative connection between the concepts, while co-occurrences below the threshold are considered random [50].

Conducting associative searches, constructing and analyzing the associative network, verbal associations, and contexts with reactions enabled the identification of implicit assessments by residents regarding the situation’s development, which the actors either did not want or could not express explicitly.

**Figure 4:**
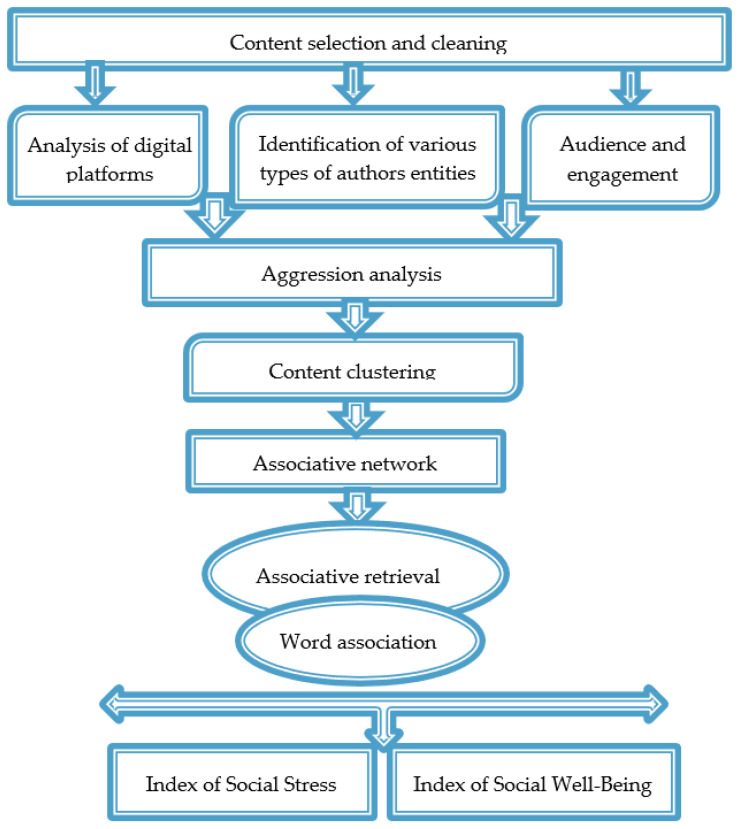
Algorithm 2.

Analyzing the dynamics of aggression, implicit opinions and assessments, and deriving social stress and well-being indices allowed for forecasting the development of the situation surrounding the project (Figure 4).

### 2.3. Tools

To collect data, Brand Analytics was used (https://br-analytics.ru/; Brand Analytics, Moscow, Russia).

The verbal content was analyzed using the neural network technology TextAnalyst 2.32 developed by one of the article authors, A.A. Kharlamov (http://www.analyst.ru; Scientific and Production Innovation Center “MICROSYSTEMS” (NPIC MICROSYSTEMS), Moscow, Russia).

For data analysis and interpretation, GPT-3.5, GPT-4, and GPT-4o were also utilized (https://chatgpt.com; OpenAI, San Francisco, CA, USA).

For visual analytics, the Tableau platform was used (https://www.tableau.com/; Salesforce, Inc., San Francisco, CA, USA).

## 3. Results

### 3.1. Algorithm 1

**Figure 5:**
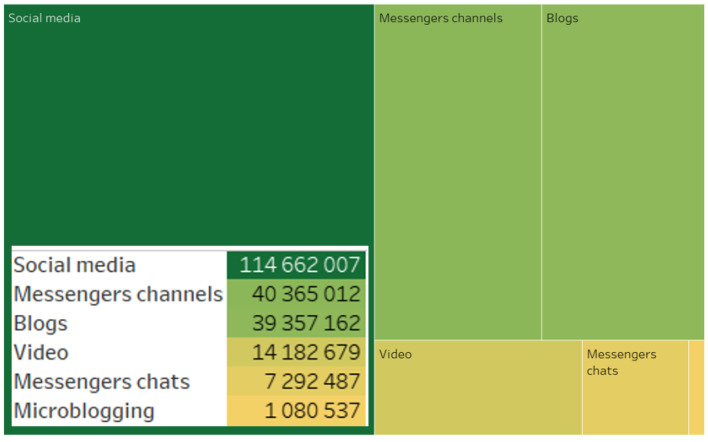
Types of sources.

After data collection and cleansing, the digital platforms where relevant content was generated were analyzed. The authors who generated content related to the project predominantly preferred using social media, as well as messenger channels and blogs (Figure 5).

**Figure 6:**
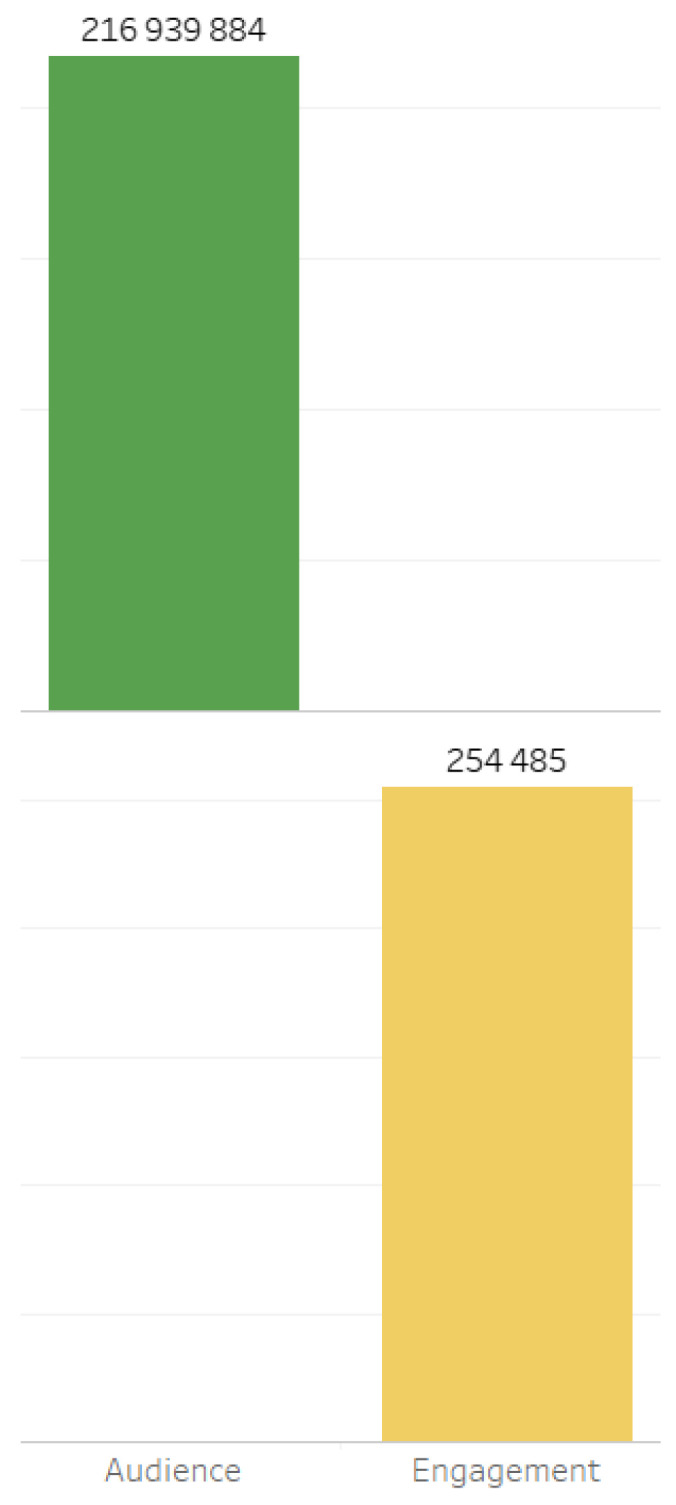
Audience and engagement metrics.

Audience and engagement indicators are presented in Figure 6.

**Figure 7:**
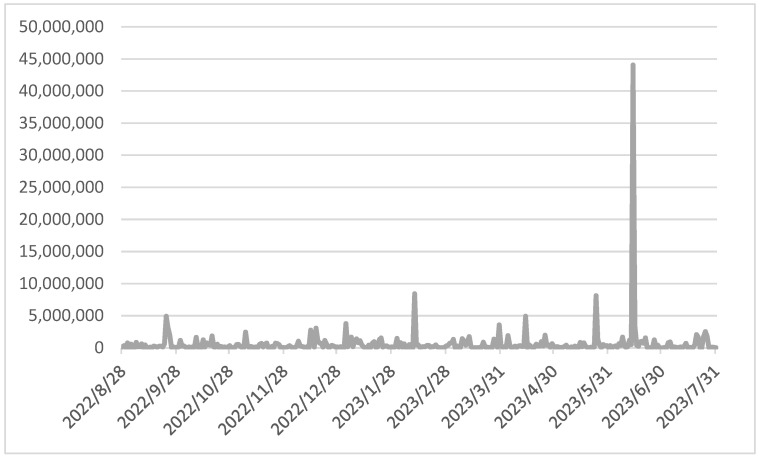
Dynamics of audience indicators.

**Figure 8:**
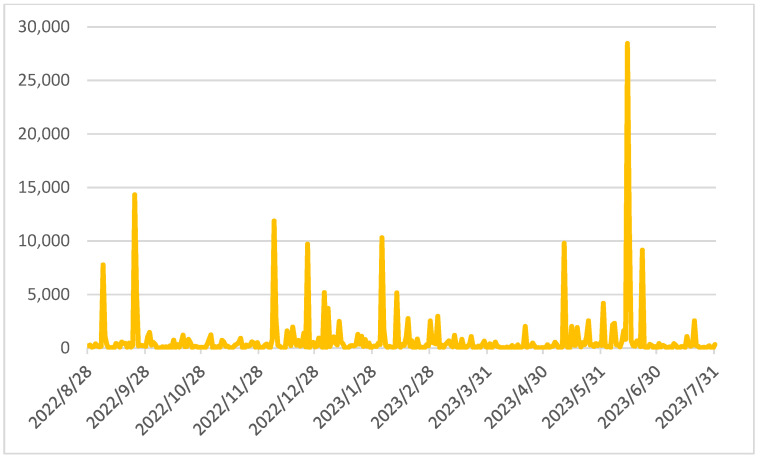
Dynamics of engagement indicators.

The dynamics of audience and engagement indicators are shown in Figure 7 and Figure 8**.** The reasons for the significant increase in audience and engagement indicators on 14 June 2023 were caused by information about the citizens’ vote, which was initiated by the city authorities, regarding the choice of designations for the new transport line on the city maps.

**Figure 9:**
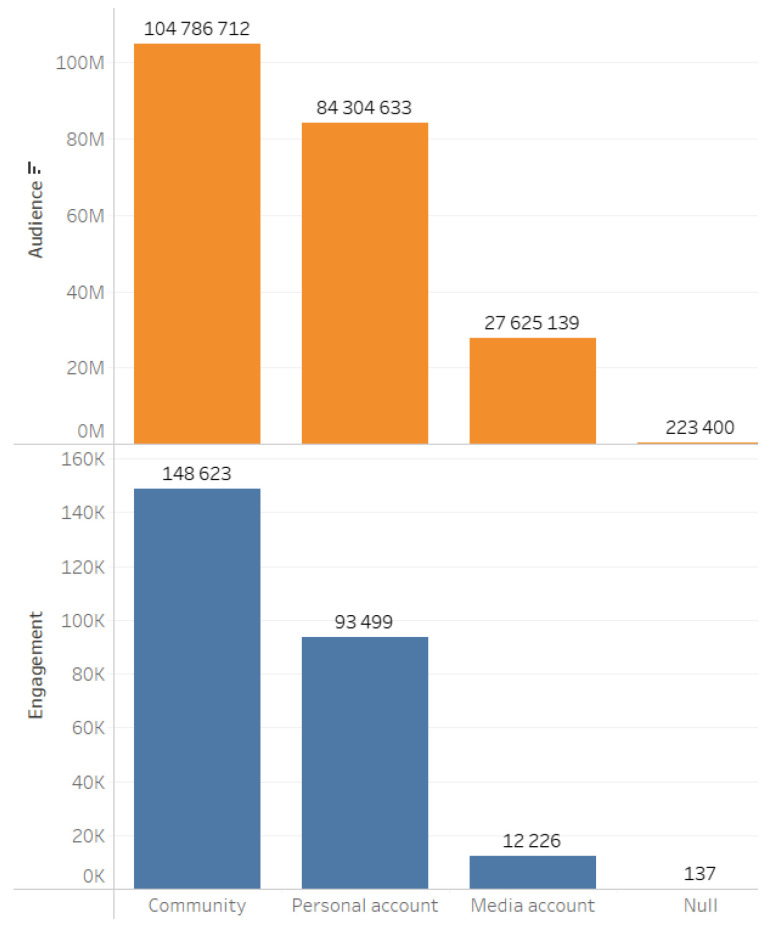
Identification of various types of author’s accounts.

Next, we identified various types of author accounts that generated content and participated in discussions related to the construction of the first phase of the project (Figure 9).

**Figure 10:**
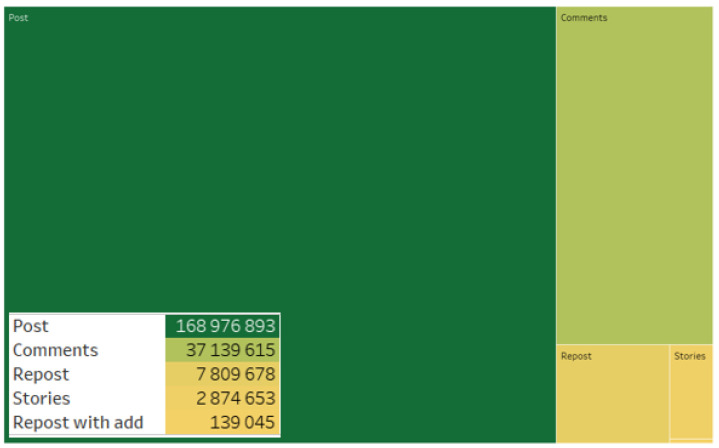
Types of messages in the consolidated database.

It should be noted that posts predominate in the consolidated database of analyzed content (Figure 10).

**Figure 11:**
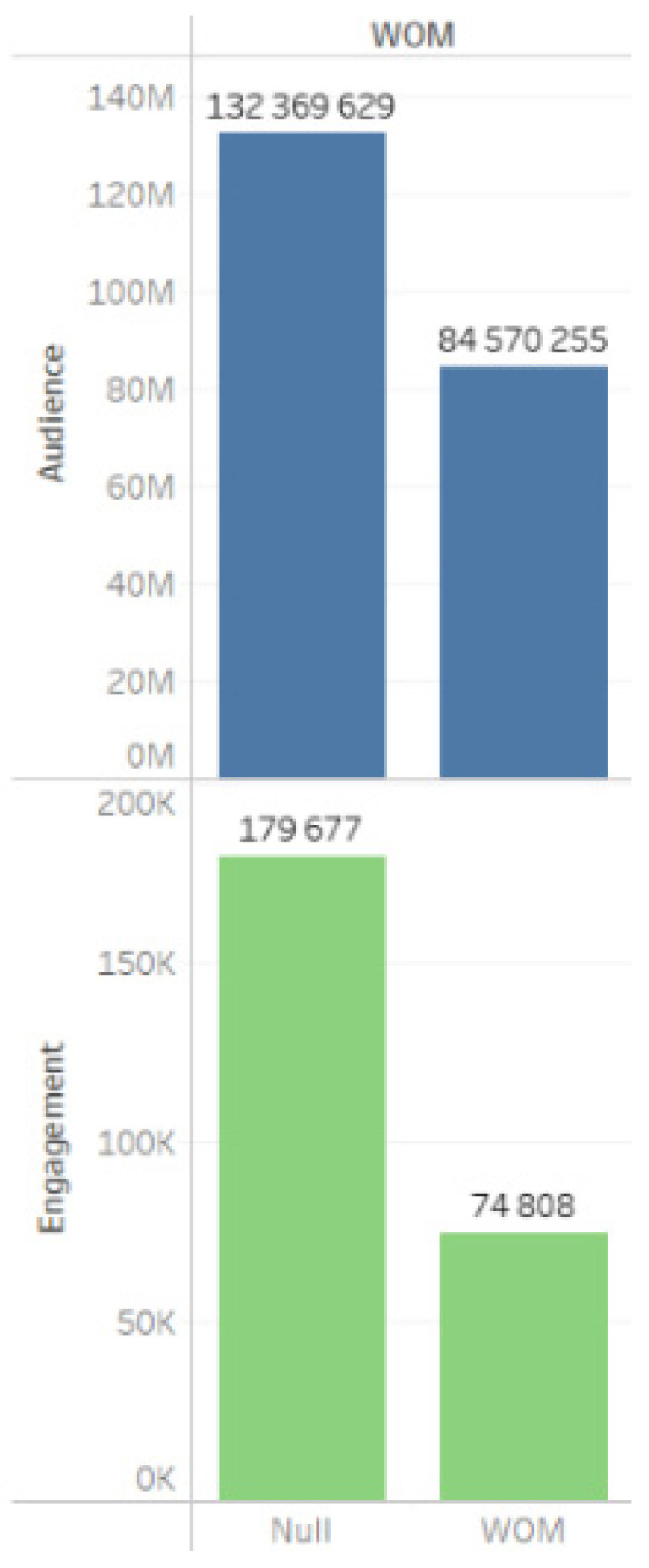
WOM messages.

**Figure 12:**
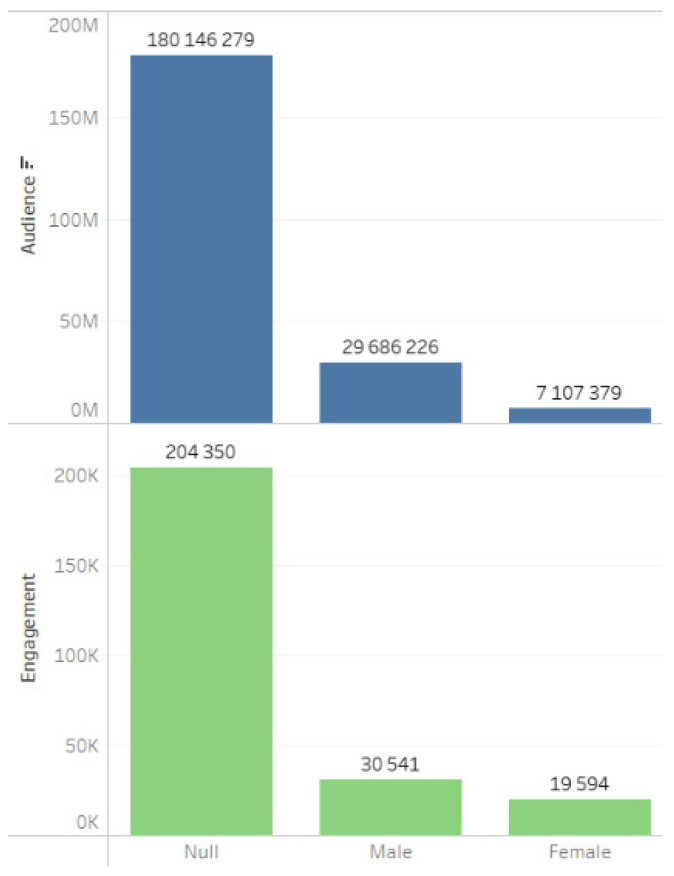
Audience and engagement by gender.

Indicators of WOM messages (word of mouth), which depict user opinions about the project based on personal experiences, written in the first person and addressed in dialogue to an initially trusting interlocutor, are presented in Figure 11. The data indicate that men, comprising the majority of the audience, were most actively engaged in discussing the issues surrounding the construction of RAL (Figure 12).

For analyzing the perception of the project implementation by the actors, the study of sentiment holds particular significance.

**Figure 13:**
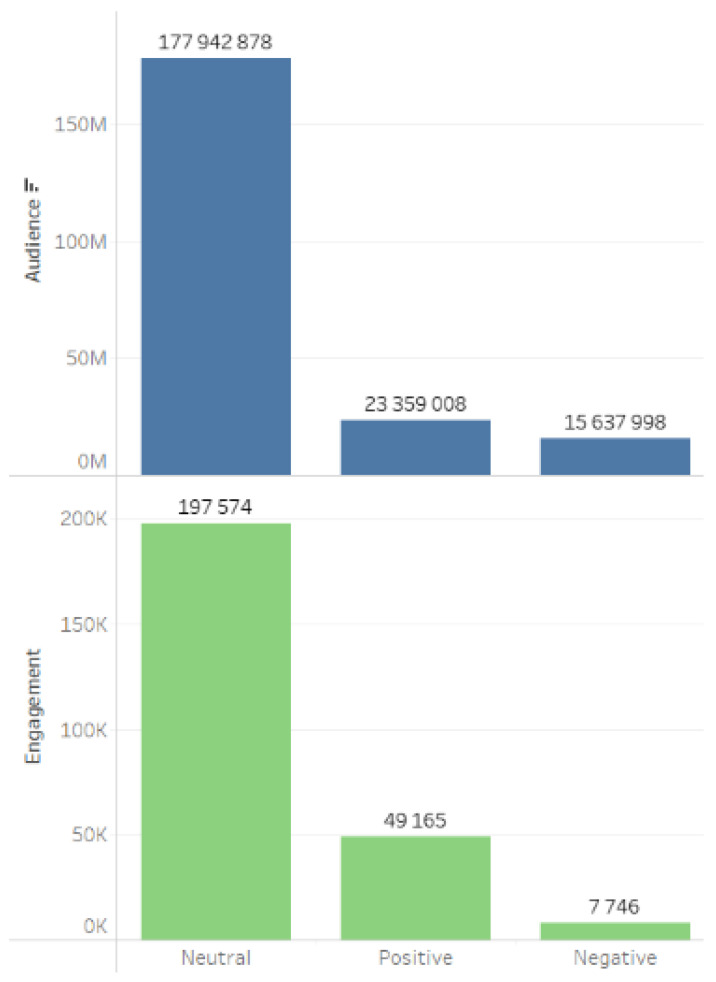
Content tonality.

The predominant sentiment in the consolidated databases dedicated to the implementation of the project is neutral (Figure 13).

**Figure 14:**
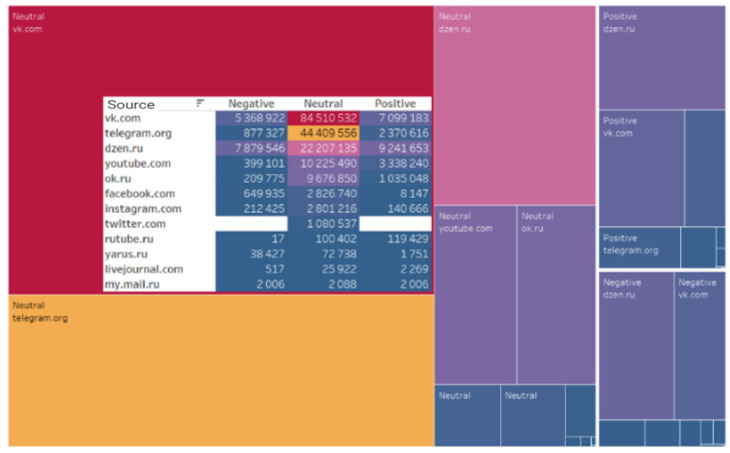
Tonality of sources.

The prevailing neutral sentiment of users toward the project is corroborated by an analysis of the sentiment across digital platforms where the content was generated (Figure 14).

**Figure 15:**
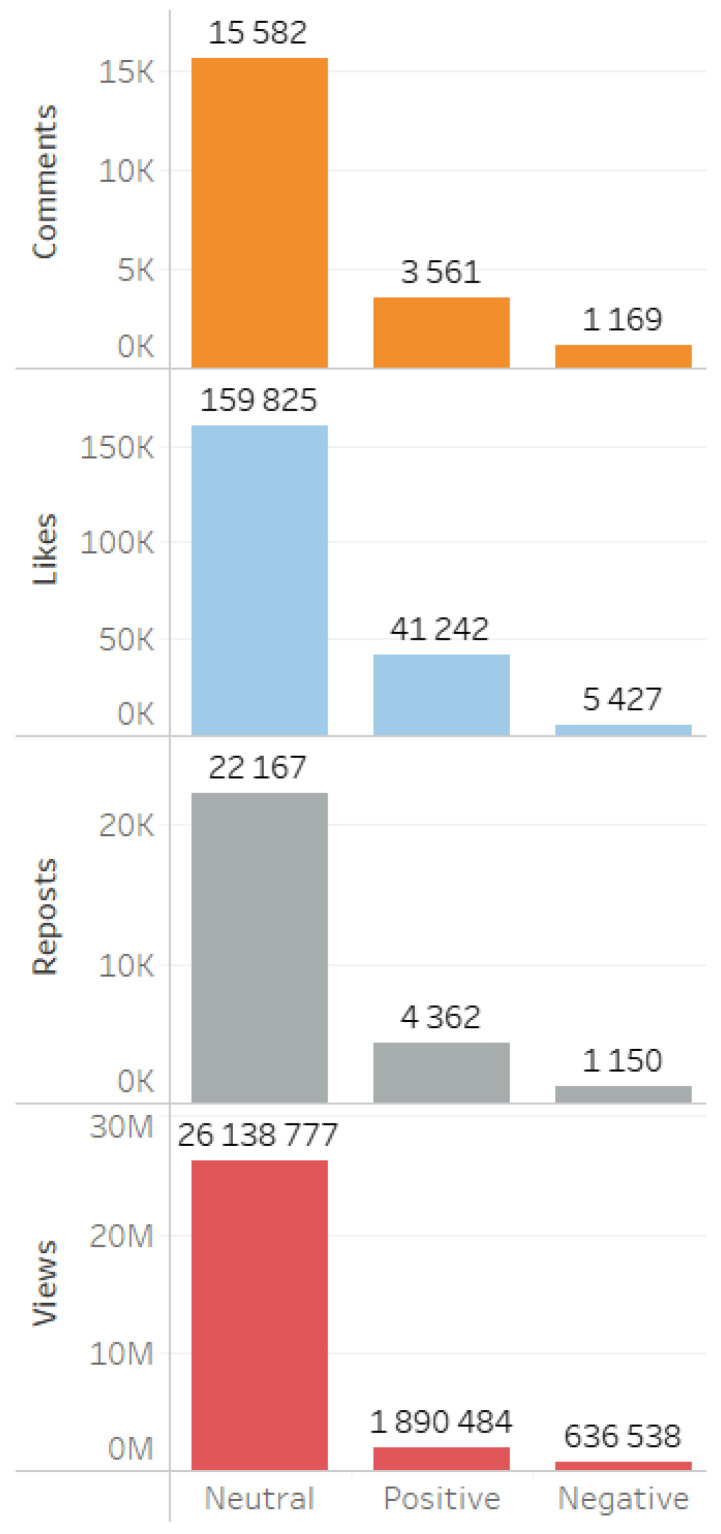
Tonality of digital footprints in the summary database.

Analysis of digital footprints indicated a predominance of neutral values in comments, likes, reposts, and views (Figure 15).

Neural network analysis of information situation data, user-generated online content surrounding the construction of the first section of the project, content clustering, sentiment analysis, and an analysis of the semantic network core have allowed for the formulation of a content rating and the determination of residents’ neutral attitude toward the construction of the first section of the project. It is specifically the analysis of the semantic network core that enables the identification and analysis of the most significant semantic accents for the actors, expressed explicitly, leading to the conclusion of a neutral perception of the situation.

For predictive analytics of the situation’s development, messages within the negative cluster are particularly significant as they reflect explicit themes related to the project that are relevant to the citizens. Negative semantic accents can, under certain conditions, act as triggers for the situation’s development.

The data analysis allowed the formation of a ranking of key negative topics that are reflected in the media space and are connected to the construction of the first section of the project. Various aspects were considered in the ranking (presence of negativity, audience size, engagement), and specific digital platforms and the consolidated database were analyzed.

- Negative cluster topic with the largest audience on Dzen (audience: 1,445,725): The introduction of new dedicated lanes for public transport and the expansion of paid parking zones are associated by actors with the construction project.
- Negative cluster topic with the largest audience on VKontakte (audience: 1,039,634): Residents believe that city authorities are dedicating too much attention and resources to developing the city’s transport system at the expense of other issues.
- Negative cluster topic with the largest audience on YouTube (audience: 258,000): Delays in the construction timeline.
- Negative cluster topic with the largest audience on Telegram (audience: 70,890): Restrictions on public transport movement due to the active construction phase.

The topics from the negative cluster with the largest audience in the consolidated database are as follows:

- Traffic restrictions due to construction work (audience: 1,121,375).
- Destruction of the city’s green zones during construction (audience: 382,900).
- Poorly planned routes and connections of new transport lines (audience: 40,035).

### 3.2. Algorithm 2

The research design, in accordance with Algorithm 2, aimed to identify key themes through the formation of a thematic structure and subsequent summarization. To analyze the perception of the actors, an analysis of digital aggression directed toward project implementation was conducted, along with an examination of the associative network and word associations, which enabled the analysis of implicit information and hidden evaluations and opinions of the users.

**Figure 16:**
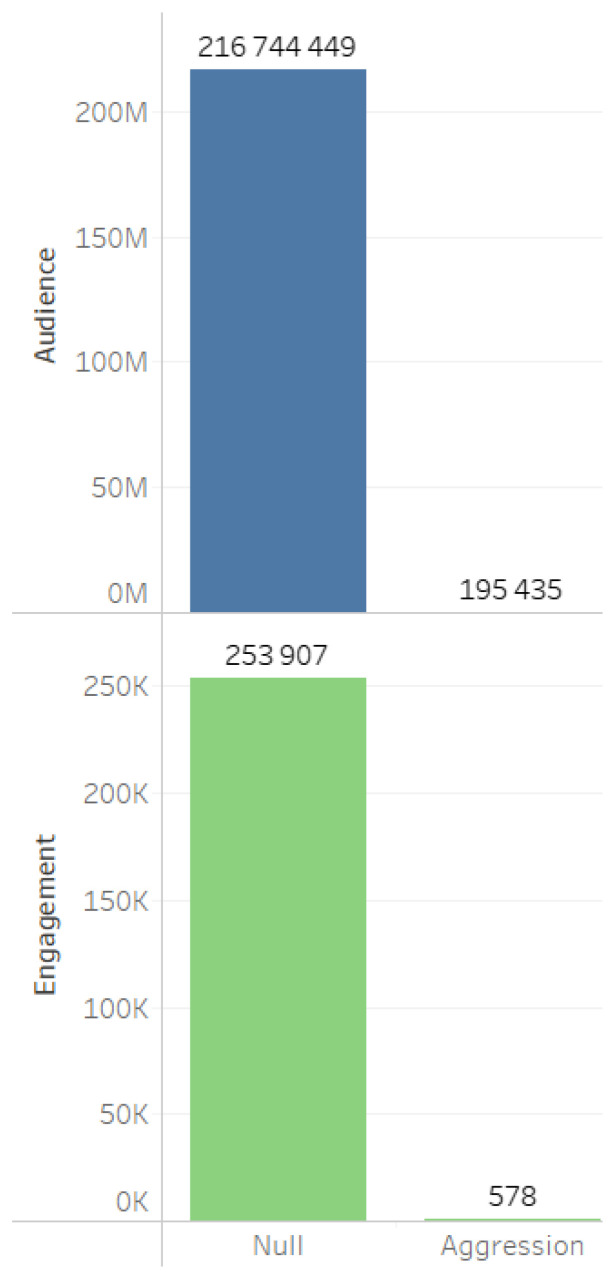
Aggression in content.

An analysis of aggression represented in the content and digital footprints of actors more vividly reflects user opinions and evaluations. It is notable that the database is characterized by minimal levels of digital aggression (Figure 16).

**Figure 17:**
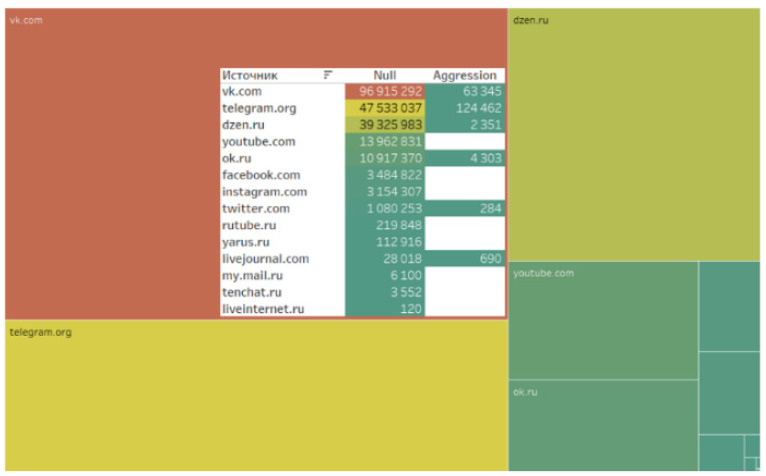
Aggression from various sources.

**Figure 18:**
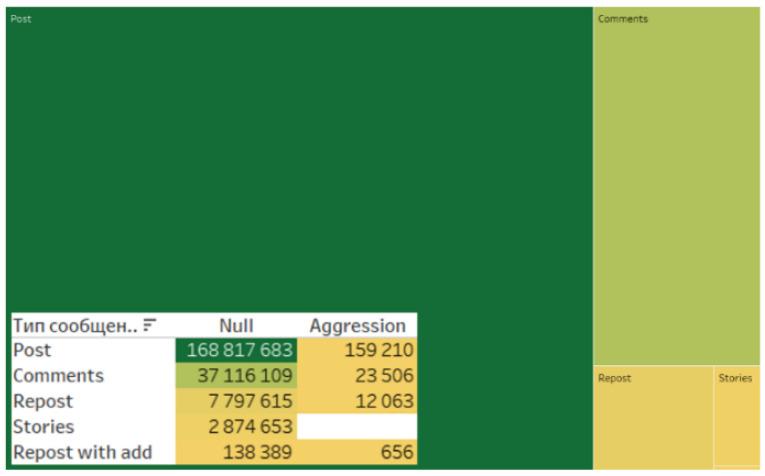
Aggression in different types of messages.

**Figure 19:**
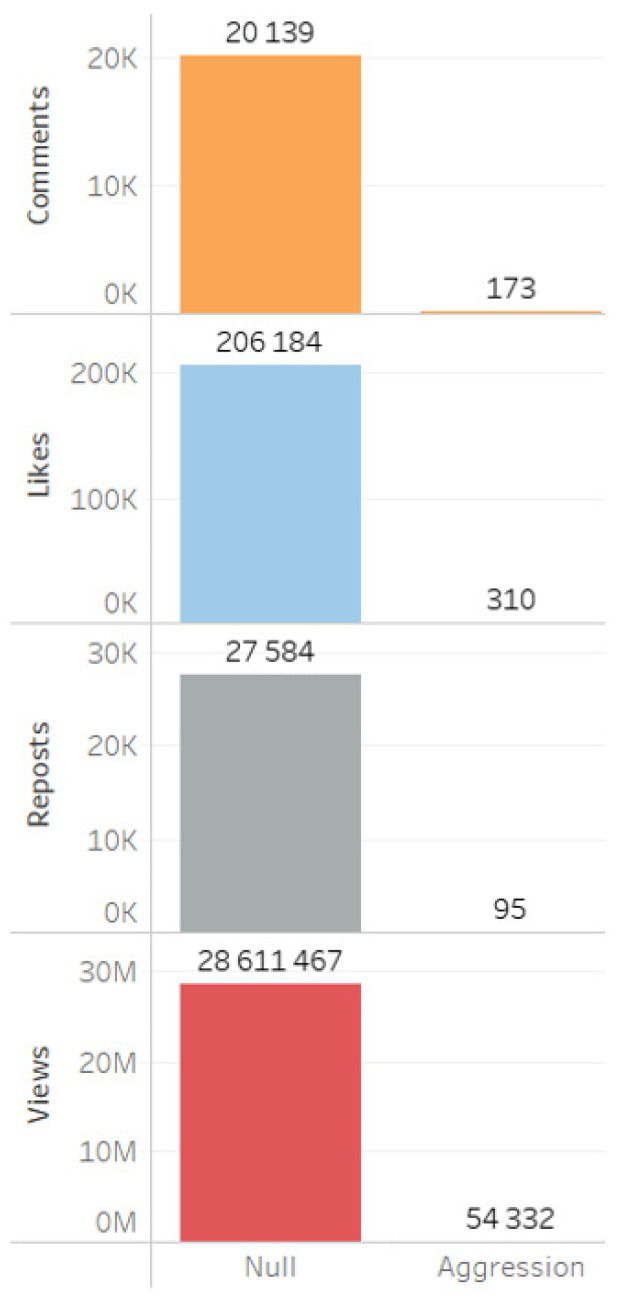
Aggression in digital footprints.

A minimal level of aggression across different sources (Figure 17), message types (Figure 18), as well as the absence of aggression in digital footprints (Figure 19) corroborate the data indicating the neutral stance of actors toward the project and the lack of social tension surrounding its implementation.

Ranking of Topics with Aggression that Reached the Largest Audience in the Consolidated Database:

1. Construction work significantly reduces the quality of life for residents, destroys old neighborhoods, and complicates city navigation (audience: 4,780,036).
2. Restrictions on public transport operations due to construction (audience: 1,121,375).
3. Destruction of part of an old cemetery during construction (audience: 678,158).
4. Residents believe that technical and safety requirements are being violated during construction (audience: 458,611).
5. Details of the voting procedure for the color of new transport lines on city maps, where the color selection caused an aggressive reaction among some residents (audience: 458,611).
6. Residents feel that city authorities are overly focused on transport projects while neglecting other city issues, such as homelessness (audience: 20,465).

A comparison of the results of the core analysis of the negative semantic cluster and the aggression cluster showed that actors’ dissatisfaction arises from objective inconveniences related to construction work.

The absence of potential triggers capable of destabilizing the situation and causing social tension was confirmed by the analysis of associative networks, verbal associations, and contexts with reactions.

Extraction of the associative network, conducting associative searches, and analyzing verbal associations enable the analysis of implicit evaluations that users do not want or cannot express openly, thus allowing the study of genuine opinions characterizing the perception of a specific situation.

Final Stage of Research under Algorithm 2: Formation of Social Stress and Well-Being Indices.

**Figure 20:**
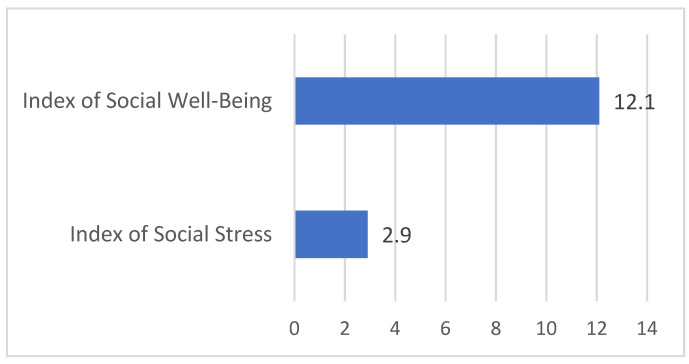
Indicators of social stress and well-being indices.

The results of the social tension analysis around the construction of the first section of the analyzed project from 28 August 2022, 00:00, to 31 July 2023, 23:59, showed the presence of social stress with a low index of 2.9 and a low social well-being index of 12.1 (Figure 20).

The social stress and well-being indices are crucial indicators of the public’s emotional perception of ongoing urban projects. Monitoring these indices allows for the assessment of social tension and general well-being among residents, providing a basis for predictive analytics regarding the development of urban situations.

- Social Stress Index: Measures the level of social tension and the prevalence of stress-inducing factors in public discourse. A high social stress index indicates increased public unrest and potential for conflict.
- Social Well-Being Index: Reflects the overall positive sentiment and contentment among residents. A high well-being index suggests a favorable perception of urban development and low levels of social stress.

These results suggest that while there is some social stress associated with the project, it remains relatively low. Similarly, the social well-being index is low, reflecting a moderate level of public satisfaction with the project. Continuous monitoring and proactive measures are recommended to ensure these indices improve over time, fostering a more positive public perception and reducing potential conflicts.

## 4. Discussion

The increasing interest in smart city technologies highlights the importance of real-time analytics on large volumes of data, modeling and predicting the development of situations across various domains, and verifying results obtained through different methodological approaches.

Algorithms and tools that combine verification and learning are of increasing interest to scientists and practitioners in a variety of fields. Problems leveraging applications of formal methods, verification, and validation, as well as adaptation and learning, were discussed in the 11th International Symposium, ISoLA 2022, Rhodes, Greece, 22–30 October 2022 [52]. The results of computer-aided verification research (AI verification, concurrency and blockchain, hybrid and cyber–physical systems, security, synthesis, etc.) are presented in [53]. Tools and Algorithms for the Construction and Analysis of Systems are described in [54]. Results of developments in web-based technologies and mobile applications, which have facilitated a dramatic growth in the implementation of new techniques, such as cloud computing, edge computing, big data, pervasive computing, Internet of Things, security and privacy, blockchain, Web 3.0, and social cyber–physical systems, can be found in [55]. During the First International Conference on Bridging the Gap between AI and Reality, AISoLA 2023, which took place in Crete, Greece, in October 2023, issues related to the nature of AI-based systems; ethical, economic, and legal implications of AI systems in practice; ways to make controlled use of AI via the various kinds of formal methods-based validation techniques; dedicated application scenarios, which may allow certain levels of assistance, etc., were discussed.

In modern studies, assessing user perceptions is usually carried out using sentiment analysis. Researchers discuss state-of-the-art work on incorporating artificial intelligence models, particularly deep learning techniques, for intelligent sentiment analysis applications. The authors assert that emotions and sentiments are emerging as critical human factors for understanding user-generated semantics and perceptions from the vast volume of user-generated data. Sentiment analysis is highlighted as a significant breakthrough technology capable of automatically analyzing human emotions in data-driven applications [56].

Valentino Sangiorgio, Luis G. Vargas, Fabio Fatiguso, and Francesco Fiorito have developed new approaches for multi-criteria analysis in construction. Their work includes classic multi-criteria analysis methods such as Analytic Hierarchy Processes and the Simos-Roy-Figueira method, as well as discussions on Augmented Reality (AR) in decision-making. The authors present a novel tool to investigate users’ perceptions, leveraging an interactive real-world environment combined with traditional methods to provide extensive visual information [57].

In this study, it was proposed that the results be verified using two algorithms in order to increase the reliability of recommendations for decision-making. The procedures selection and analysis of the topic structure and summarization made it possible to highlight the main topics that turned out to be important for residents; comparison of the results of semantic analysis and analysis of the associative network also made it possible to confirm the verification of the results obtained by parallel methods.

It should be noted that researchers pay special attention to studying associative relationships through the paradigm of verbal associations (WA) [58]. With the help of implicit association tests (IAT), it helped study implicit social cognition, subconscious motivations, attitudes toward the presented stimulus, as well as automatic associations for subjects who prefer not to demonstrate at a conscious level (see, for example, Project Implicit (https://www.projectimplicit.net/ (accessed on 28 March 2024))).

The potential of associations in analyzing various types of network data has also already demonstrated its effectiveness [59]. In this study, lexical association analysis was conducted on clusters identified during the analysis of digital aggression, which allowed not only drawing conclusions about the perception of the situation by the users, identifying the most frequent associations characterizing the actors’ attitude toward the subject of analysis, but also revealing implicit knowledge.

The main limitation of the conducted research is that only user-generated verbal content and digital footprints were used as material. However, the role of integrated forms is increasing in modern media space. For a more accurate analysis of the actors’ perceptions of virtual interaction, multimodal formats should be employed. The development of a multimodal approach is the next stage in the research.

The algorithms for validating and studying social network data require further refinement and adaptation to specific practical projects. It is evident that the analysis and interpretation of large volumes of social network data require an interdisciplinary approach, integrating methods from various disciplines (computer science, mathematics, statistics, linguistics, psycholinguistics, sociology, psychology, etc.).

## 5. Conclusions

During the study, algorithms for analyzing and interpreting social media data to assess citizens’ opinions in real time and for verifying and examining social media data were used to analyze social tension and predict the development of situations during the implementation of urban projects.

The testing of the developed algorithms was carried out using the example of the implementation of a city project in the field of the development of a transport system.

The first algorithm was based on sentiment analysis and semantic network analysis, while the second algorithm focused on aggression analysis and associative search.

The results of the data analysis using both algorithms showed identical outcomes. Negative semantic and informational accents in user perceptions were identified and analyzed, which could potentially lead to conflicting situations, along with implicit and explicit evaluations and opinions. Sentiment analysis and aggression analysis of user-generated content and digital footprints were also conducted. Indices of social stress and well-being were derived. This led to the conclusion of a neutral perception among users and the absence of social tension surrounding the implementation of the project. Recommendations were also developed to mitigate potential conflicts and eliminate sources of social tension for decision-making purposes.

Subsequent developments regarding the project have validated the accuracy of the obtained results.

## Figures and Tables

**Table 1:** Quantitative characteristics of the database.

Activities	Number
Audience	216,939,884
Engagement	254,485
Loyalty	1,9

**Table 2:** Data characteristics.

Digital Platform	Audience	Tokens
VKontakte	96,978,637	13,728,752
Telegram	45,657,499	2,944,209
Dzen	39,328,334	2,313,912
Youtube	13,962,831	1,222,741

## Data Availability

Publicly available datasets were analyzed in this study.

## Supplementary Materials

The following supporting information can be downloaded at: https://www.mdpi.com/article/10.3390/s24154810/s1, Table S1: Additional characteristics of the database.
